# From Bioinactive ACTH to ACTH Antagonist: The Clinical Perspective

**DOI:** 10.3389/fendo.2017.00017

**Published:** 2017-02-08

**Authors:** Chiraz Ghaddhab, Jean-Marc Vuissoz, Johnny Deladoëy

**Affiliations:** ^1^Endocrinology Service and Research Center, Sainte-Justine University Hospital Center, Department of Pediatrics, Université de Montréal, Montreal, QC, Canada; ^2^Division of Pediatric Endocrinology, University Children’s Hospital Basel (UKBB), University of Basel, Basel, Switzerland

**Keywords:** stress, cortisol, adrenals, ACTH, antagonist

## Abstract

The adrenocorticotropic hormone (ACTH) is a pituitary hormone derived from a larger peptide, the proopiomelanocortin (POMC), as are the MSHs (α-MSH, β-MSH, and γ-MSH) and the β-LPH-related polypeptides (Figure 1A). ACTH drives adrenal steroidogenesis and growth of the adrenal gland. ACTH is a 39 amino acid polypeptide that binds and activates its cognate receptor [melanocortin receptor 2 (MC2R)] through the two regions H_6_F_7_R_8_W_9_ and K_15_K_16_R_17_R_18_P_19_. Most POMC-derived polypeptides contain the H_6_F_7_R_8_W_9_ sequence that is conserved through evolution. This explains the difficulties in developing selective agonists or antagonists to the MCRs. In this review, we will discuss the clinical aspects of the role of ACTH in physiology and disease, and potential clinical use of selective ACTH antagonists.

## From the Concept of “Stress” to the Discovery of Adrenocorticotropic Hormone (ACTH)

Adrenocorticotropic hormone and cortisol are nowadays associated with the physiological response to stress. Historically, the concept of stress was first introduced in 1936 by Hans Selye (1). It was first called the “general adaptation syndrome” and later renamed by Selye as “stress response.” Stress results from the balance between the stressor, an agent that produces stress at any time, and the body’s adaptive response to it. His experiments on rats in 1936 have shown that a stressor often alters the adrenal cortex, the immune system, and the gut. Indeed, rats exposed to various nocuous chemical or physical stimuli had a hypertrophy of the adrenals, an involution of the lymphatic nodes and developed gastric erosions (2).

In the 1930s–1940s, Selye performed extensive structure–function studies, resulting in the first classification of steroid hormones, e.g., corticoids, testoids/androgens, and folliculoids/estrogens (3, 4). During those years, he recognized the respective anti- and pro-inflammatory actions of gluco- and mineralocorticoids (named by Selye) in animal models, several years before demonstration of anti-rheumatic actions of cortisone and ACTHs in patients. In 1935 and 21 years after having isolated thyroxine in crystalline form, Kendall isolated and identified the structure of cortisone, and in 1936, Reichstein identified the structure of cortisol. In 1948, Hench and Kendall demonstrated that cortisone and ACTH exert a profound anti-inflammatory effect in patients with rheumatoid arthritis. In 1950, Hench, Kendall, and Reichstein were awarded the Nobel Prize in Physiology and Medicine for these discoveries. In the same year, Harris established that ACTH secretion involves “neuronal control *via* the hypothalamus and the hypophyseal portal vessels of the pituitary stalk” (5). ACTH was first isolated in 1943 and then synthesized in the 1960s and 1970s by different groups (6–9). Next, the amino acid sequences of ACTH from four mammalian species were elucidated between 1954 and 1961 by three groups of Bell, Li, and Lerner (6, 10, 11). In 1955, Guillemin (a former student of Selye’s) and Rosenberg demonstrated in rats the existence of CRF, the hypothalamic factor that allows ACTH release (12).

## ACTH Structure and Function

**Figure 1:**
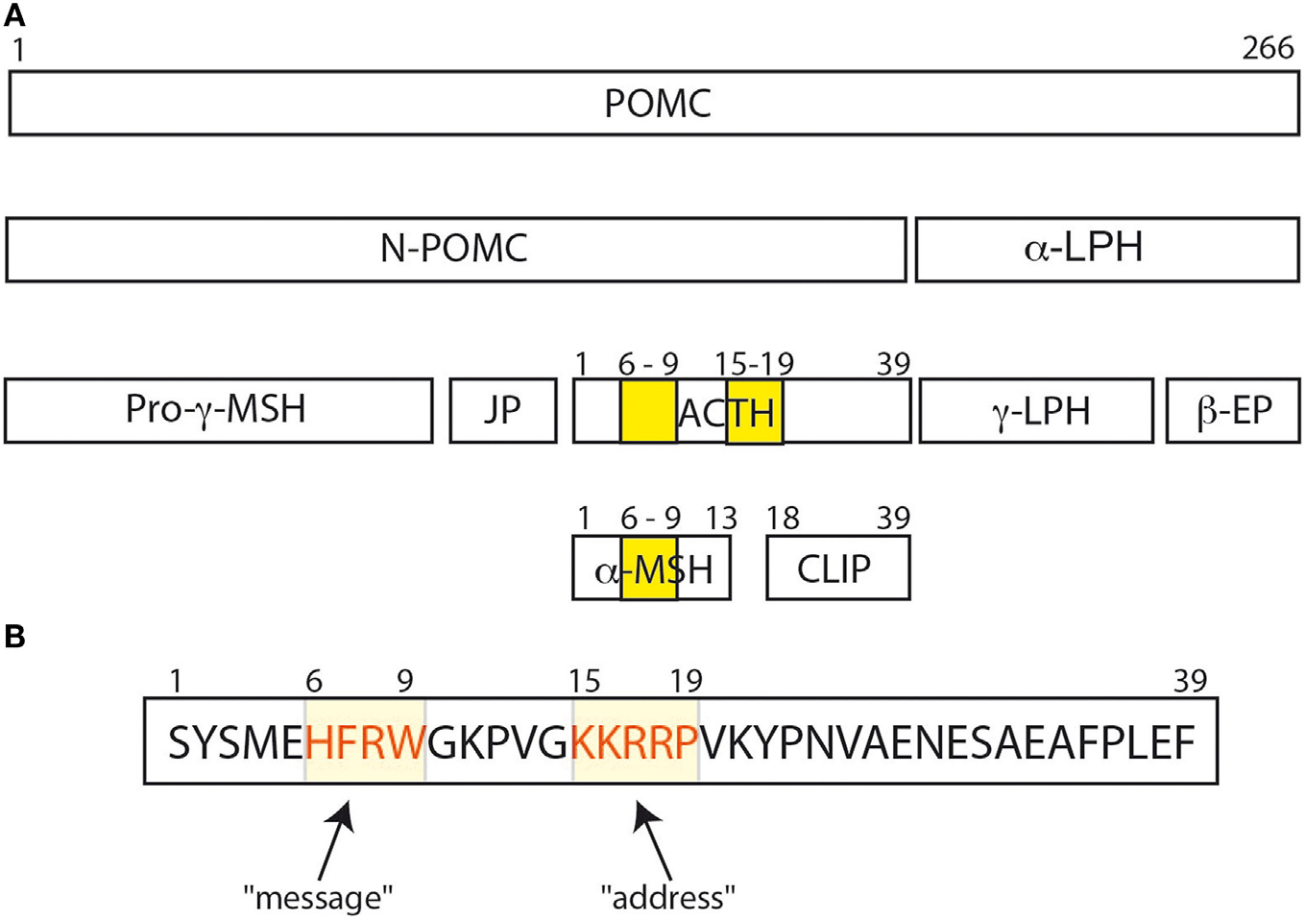
**(A)** The POMC protein and its various peptide cleavage products: the yellow bands correspond to the amino acid sequences His^6^Phe^7^Arg^8^Trp^9^ and Lys^15^Lys^16^Arg^17^Arg^18^Pro^19^. His^6^Phe^7^Arg^8^Trp^9^ is important for binding and signal transduction of α-MSH. **(B)** His^6^Phe^7^Arg^8^Trp^9^ sequence is important for adrenocorticotropic hormone (ACTH) signal transduction and for ACTH binding to melanocortin receptor 2 (MC2R) (26), and was called the “message” sequence (13). The amino acids Lys^15^Lys^16^Arg^17^Arg^18^Pro^19^ was initially defined as the “address” sequence allowing specific ACTH binding to MC2R (27).

**Figure 2:**
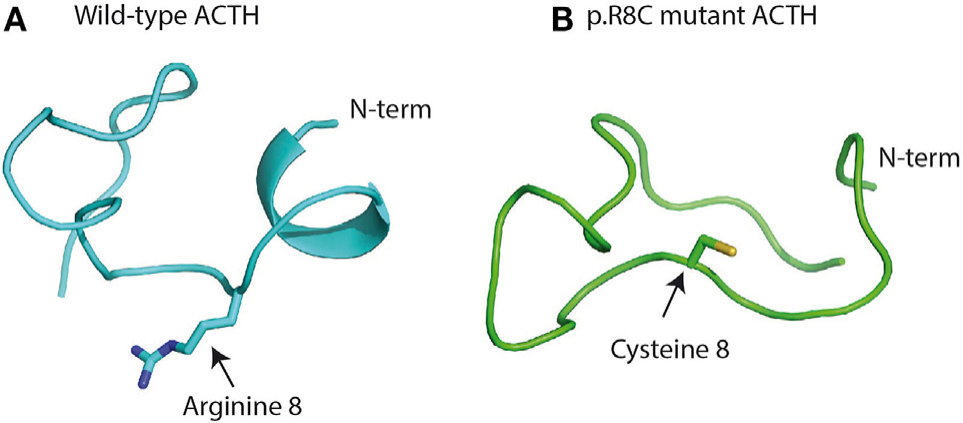
**Structure of wild-type (A) and mutant adrenocorticotropic hormone (ACTH) (B) after a 100-ns molecular dynamics simulation in implicit water (28, 29): (A) to retain full bioactivity, the N-terminal “message” sequence has to conserve its helical secondary structure as postulated by Schwyzer 40 years ago (13)**. **(B)** Naturally occurring bioinactive ACTH mutation (p.R8C) disrupts the helical conformation and hampers proper binding and activation of the melanocortin receptor 2 (26).

Adrenocorticotropic hormone, adrenocorticotropin, corticotropin, or ACTH, transmits information from the anterior lobe of the pituitary to the adrenal cortex. ACTH is a linear non-atriacontapeptide with species differences in the COOH terminal two-third of the molecule (13). Initially, an analysis of the ACTH crystal structure was still lacking. In the early 1970s, different polypeptides were isolated from different lobes of the vertebrate pituitary (14). Lowry showed that ACTH and β-lipotropin (β-LPH) shared the same amino acid motif H_6_F_7_R_8_W_9_ even if they have different functions (14, 15) (Figure 1). α-MSH and β-MSH were found to be embedded within the primary sequences of ACTH and β-LPH, respectively, in the melanocorticotropic cells (in the intermediate pituitary) (16). A few years later, the prediction that ACTH, the MSHs, and the β-LPH-related polypeptides were derived from the same precursor (17) was confirmed by the cloning of the proopiomelanocortin gene (POMC) mRNA (18). Initially, Schwyzer and Sieber suggested that ACTH is a linear polypeptide (19). Later, Schiller showed that residues 10–21 have a flexible conformation (20). Squire and Bewley had already suggested that a small region close to N-terminus had a tendency to form a helical structure (21). Low et al. have confirmed that a helical structure is produced in a solvent, the trifluoroethanol (22). It was suggested that this conformational change could occur when ACTH binds its receptor, to ensure a kinetically and a thermodynamically optimal hormone–receptor contact (23, 24). Later, Hruby et al. showed that the N terminal region of ACTH, between residues 4 and 10, adopts a β-turn conformation (25). Molecular modeling supports this hypothesis, given the presence of an N-terminal helical structure in the wild-type ACTH model (Figure 2A). This helical secondary structure is disrupted by validated inactivating mutations, which suggests that it is essential for ACTH bioactivity (Figure 2B).

Due to different bioassay systems, several research teams screened the biological activity of C terminally truncated analogs of ACTH (1–39) (30). ACTH (1–24) was as potent as ACTH (1–39) to induce glucocorticoid production by adrenal cells in culture (31), while ACTH (1–16) was unable to do so. It was concluded that the critical portion of ACTH for activating adrenal cells resided between residues 17 and 24 (31). However, the analog ACTH (11–24) was unable to induce glucocorticoid production and was even an antagonist at high concentrations (31). Fauchère et al. showed that dimers of ACTH (11–24) and ACTH (7–24) were potent antagonists, whereas ACTH (1–24) dimers remained agonists but were 70 times less potent when compared to its monomeric ACTH (1–24) (32–34).

Altogether, Schwyzer concluded that residues 17–24 were important for recognition of ACTH by its adrenal receptor, and he coined the term “address sequence” to indicate that these residues allowed the ligand to find the cognate receptor (Figure 1B) (13, 27). As Costa et al. suggested, the position 20 should be included in the address site (35). To activate the adrenal receptor, a “message” region is needed. Several studies suggested that the H_6_F_7_R_8_W_9_ motif should be considered as the “message sequence” (13) (Figure 1B). Indeed, both address and message sequences are necessary to activate the adrenal receptor: the address sequence interacts with the adrenal receptor and then the message sequence enters in contact with the receptor to induce the conformation change in the receptor that activates the adrenal cell (27). This could explain why α-MSH could not activate adrenal cells, even if it has the H_6_F_7_R_8_W_9_ motif. On the other hand, Schwyzer considered also the effect of analogs of ACTH (1–39) on the activation of melanocytes. All analogs of ACTH (N-terminally as well as C terminally truncated forms) were able to activate melanocytes as long as the H_6_F_7_R_8_W_9_ motif was intact (13, 27), and the H_6_F_7_R_8_W_9_ motif is probably both the message and the address sequence in these cells.

In the 1990s, five melanocortin receptor (MCR) genes were identified in the genome of mammals, tetrapods, birds, bony fish, and cartilaginous fish (36–39). MC1R mediates pigmentation, MC2R activates glucocorticoid biosynthesis in the adrenal cortex, MC3R and MC4R influence metabolic homeostasis in the central nervous system and periphery (40), and MC5R regulates sebaceous gland secretions (41). The MCRs belong to the rhodopsin family of G protein-coupled receptors (GPCRs) (37, 42). They induce intracellular cyclic AMP production following activation by ligands (43, 44). ACTH (1–39) or α-MSH can activate MC1R, MC3R, MC4R, and MC5R (37), but MC2R can only be activated by ACTH and not by α-MSH or other analogs (45). The MCR genes have now been cloned and expressed in cell lines, allowing *in vitro* binding and activation assays of ACTH analogs against all MCRs. Recently, it has been shown that MC2R requires the presence of the melanocortin receptor-associated protein (MRAP) to be expressed and active at the cell surface (46–49).

Although ACTH and α-MSH both have the H_6_F_7_R_8_W_9_ motif, only ACTH can activate MC2R. This raises the question of differences between the H_6_F_7_R_8_W_9_-binding site in MC2R when compared to the other MCRs. Due to a crystal structure of the H_6_F_7_R_8_W_9_-binding site and site-directed mutagenesis analyses of human MC4R, we know that vertebrate MCRs have a common H_6_F_7_R_8_W_9_-binding site (50–53), which further confirms that the H_6_F_7_R_8_W_9_ motif is both the message sequence and the address sequence for the α-MSH. Dores hypothesized that, prior to stimulation, MC1, MC3, MC4, or MC5 receptors have a binding site that is in an open conformation for the ligand. When the ligand interacts with the receptor, it leads to a conformational change (a “docking event”) that activates the receptor (27). Therefore, the activation of MC2R may be a multistep process that requires (i) a conformational change after docking of the H_6_F_7_R_8_W_9_ motif on its MC2R-binding site and, in the second step, (ii) docking of the K_15_K_16_R_17_R_18_P_19_ motif of ACTH to activate the MC2R receptor (27, 54). This could explain the differential ligand selectivity of the MCRs. However, further analyses and experiments are necessary to confirm this hypothesis.

## Familial Glucocorticoid Deficiency (FGD) and POMC Deficiency

### Familial Glucocorticoid Deficiency

The glucocorticoid deficiency syndrome (FGD) is an autosomal recessive disorder characterized by insensitivity to ACTH action on the adrenal cortex (55), thereby resulting in glucocorticoid insufficiency with intact mineralocorticoid secretion. It may manifest during early neonatal life but can be diagnosed later during childhood. Clinical manifestations include recurrent hypoglycemia that may lead to seizures and coma, chronic fatigue, failure to thrive, recurrent infections, and skin hyperpigmentation. Typically, plasma cortisol levels are very low or undetectable without response to ACTH, while endogenous ACTH levels are very high, which clearly shows a specific resistance to the action of ACTH in these patients. The aldosterone and renin levels are usually normal and correctly match the activation of the renin–angiotensin axis. Even if congenital adrenal hyperplasia (CAH) (due to defects in the glucocorticoid synthesis pathway), congenital adrenal hypoplasia, and X-linked adrenoleukodystrophy are associated with high ACTH and low cortisol levels, they are distinct from FGD.

So far, mutations in five genes have been associated with isolated FGD and four with FGD associated with various syndromic features. In all these genetic disorders, low cortisol and a high ACTH suggest ACTH resistance. Causative genes of *isolated* FGD are the ACTH receptor (*MC2R*; also formerly defined as FGD 1) (56), the melanocortin receptor-associated protein (*MRAP*; also formerly defined as FGD 2) (46), the DAX1 transcription factor (*NR0B1*) (57), nicotinamide nucleotide transhydrogenase (*NNT*) (58–60), and mitochondrial thioredoxin reductase (*TXNRD2*) (61). Interestingly, NNT and thioredoxin reductase (TXNRD2) are crucial enzymes for the production of sufficient NADPH to maintain the redox potential in the steroid-producing cells. Four *syndromes* have been reported with FDG: (i) the AAA(A) syndrome (ACTH resistance, alacrimia, achalasia, and autonomous system dysfunction) due to *AAAS* mutations (62); (ii) the IMAGe syndrome (intrauterine growth restriction, metaphyseal dysplasia, adrenal hypoplasia congenita, and genital anomalies) due to *CDKN1C* mutations (63); (iii) a syndrome with short stature, recurrent infection due to natural killer cell deficiency, and chromosomal fragility associated with mutation of the DNA helicase, minichromosome maintenance 4 (*MCM4*) (64); and (iv) the MIRAGE syndrome (myelodysplasia, infection, restriction of growth, adrenal hypoplasia, genital phenotypes, and enteropathy) due to mutations in *SAMD9* (65).

### POMC Deficiency

Proopiomelanocortin (POMC) deficiency causes severe monogenic obesity that begins at an early age, adrenal insufficiency, red hair, and pale skin. Affected infants have a normal weight at birth, but they develop early hyperphagia that leads to excessive weight gain starting in the first year of life. During the neonatal period, patients usually present with hypoglycemic seizures, hyperbilirubinemia, and cholestasis due to secondary hypocorticism (66). Subclinical hypothyroidism was also reported. The incidence is very low, about one in 1 million. Complete POMC deficiency is an autosomal recessive disorder and is caused by homozygous or compound heterozygous loss-of-function mutations in the POMC gene on chromosome 2p23.3.

POMC is regulated by leptin and is cleaved by prohormone convertases to produce ACTH and MSH α, β, and γ. In POMC deficiency, the serum concentrations of these cleavage products are low. The red hair pigmentation, adrenal insufficiency, and obesity are caused by inability to activate the MC1, MC2, and MC4 receptors, respectively (66). In addition to complete POMC deficiency, isolated deficiency of β-MSH has been described. This isolated deficiency of β-MSH leads to severe obesity without adrenal insufficiency or red hair (67, 68).

A few years ago, we reported glucocorticoid deficiency in two unrelated patients with apparent ACTH resistance due to an unusual mutation in POMC: the p.R8C mutation in the sequence encoding ACTH and α-MSH (26). The patients (a 4-year-old girl and a 4-month-old boy) presented with hypoglycemia, low cortisol, normal electrolytes, and high ACTH. Both patients had red hair, adrenal insufficiency, and developed early-onset obesity. They were initially treated with gluco- and mineralocorticoids, but after the identification of the mutation in POMC, mineralocorticoid treatment was discontinued in both patients. Whole exome sequencing revealed that the girl was compound heterozygous for POMC mutations: one previously described null allele and one novel p.R8C mutation in the sequence encoding ACTH and α-MSH. The boy was homozygous for the p.R8C mutation. We demonstrated that even if ACTH-R8C was immunoreactive, it failed to bind and activate cAMP production in melanocortin-2 receptor (MC2R)-expressing cells. We demonstrated also that α-MSH-R8C failed to bind and stimulate cAMP production in MC1R- and MC4R-expressing cells.

Discovery of this mutation indicates that in humans, unlike rodents, the amino acid sequence H_6_F_7_R_8_W_9_ is important not only for cAMP activation but also for ACTH binding to the MC2R. Furthermore, *in silico* modeling suggested that the pR8C mutation disrupts the secondary structure of ACTH, which implies that this secondary structure may have a crucial role for M2R activation (Figure 2).

Diagnosis of complete POMC deficiency may be suspected on the basis of clinical manifestations and can be confirmed by identification of mutations in the *POMC* gene. To treat POMC-deficient patients, only hydrocortisone substitution is currently available and is required. An intranasal administration of the ACTH 4–10 melanocortin fragment did not lead to a reduction in body weight. Administration of thyroid hormone had no effect on obesity (69). Recently, two extremely obese POMC patients have been substituted with a MC4R agonist (setmelanotide), and a significant reduction of their weight has been achieved (more than 10% after 10 weeks of treatment) (70). This paves the way for treatment of this condition with specific MC4R agonists and should also prompt research into developing agonists to other MCRs such as MC2R.

## Perspectives: A Role for Specific ACTH Antagonists in Human Disease

Specific ACTH antagonists would be of great value especially to treat two conditions: CAH and primary bilateral macronodular adrenal hyperplasia (PBMAH). Moreover, MC2R antagonists could represent a valuable alternative to anticortisolic drugs in clinical management of ACTH-dependent hypercortisolism, essentially Cushing’s disease, when removal of the source of ACTH excess is impossible or incomplete.

### Congenital Adrenal Hyperplasia

Adrenocorticotropic hormone drives the synthesis and secretion of cortisol as well as androgens from the adrenal gland. In CAH due to 21 hydroxylase deficiency, excess of ACTH leads to overandrogenization. CAH is due to the enzymatic defect in the cortisol synthesis pathway, with subsequent hypocortisolism, ACTH overproduction, accumulation of androgen precursors, and adrenal gland hyperplasia. Treatment with glucocorticoids at physiological doses is life saving but is not sufficient to suppress the elevated ACTH levels and androgen overproduction. Failure to suppress excess androgens results in height acceleration, advanced skeletal maturation, and eventually leads to decreased final adult height. Other effects of androgen excess include clitoromegaly and hirsutism in females, isosexual pseudoprecocious puberty in males, and acne and deepening of the voice in both sexes. Only supraphysiological doses of glucocorticoids suppress ACTH in CAH but at the cost of hypercortisolemia with its adverse effects such as hyperglycemia, arterial hypertension, reduced growth, and osteoporosis. Since MC2R is expressed only in the adrenals, it could become a specific target. Therefore, an adjuvant drug able to specifically block the MC2R would obviate the need for supraphysiological dose of glucocorticoids in CAH and prevent the undesirable effects inherent to glucocorticoid overtreatment.

### MC2R Expressing PBMAH

Primary bilateral macronodular adrenal hyperplasia (also known as ACTH-independent macronodular adrenal hyperplasia) is a rare, sporadic disease affecting men and women with an almost equal ratio. PBMAH has a bimodal age distribution: during the first year of life (minority) when it may be associated with the McCune–Albright syndrome (71) and during the fifth decade of life (majority) (72). Occurrence of familial cases of PBMAH and involvement of both adrenal glands (even in sporadic cases) strongly suggest involvement of germline genetic predisposition in PBMAH. Indeed, inactivating mutations of the armadillo repeat containing 5 (*ARMC5*) have been reported in 25–55% of patients with sporadic PBMAH (73–75).

The most frequent clinical manifestation of PBMAH is Cushing syndrome. In PBMAH, one previously believed that glucocorticoid secretion is ACTH independent given that, most of the time, plasma ACTH levels are undetectable, and high-dose dexamethasone administration fails to suppress cortisol secretion. This notion has been revised following the demonstration of local production of ACTH. Indeed, Cheitlin et al. studied the adrenal cells from a patient with PBMAH *in vitro*; the cells were cultured on an extracellular matrix and demonstrated rapid growth and a high rate of cortisol secretion in the absence of ACTH (76). Not only the MC2R was expressed in PBMAH tissue (77) but also ACTH was abnormally expressed, further stimulating the cortisol secretion through a paracrine effect (78). These findings represent a strong incentive to treat PBMAH-associated hypercortisolism with MC2R antagonists. This contrasts with primary pigmented nodular adrenocortical disease, sporadic large benign adenomas, and adrenocortical carcinomas, which are mostly ACTH unresponsive (79).

Even if these observations suggest that specific MC2R antagonist could help patients with PBMAH, the specific structure of the ACTH produced by PBMAH cells (78) and in ectopic Cushing’s syndrome (80) might raise unexpected difficulties. Indeed, these bioactive ACTH are not detected by antibodies directed to the ACTH C terminal portion. This may result from extrapituitary sources of ACTH producing increased amounts of precursors (pre-ACTH and POMC) due to impaired POMC processing (81, 82). Reports on receptor affinity and steroidogenic potency of ACTH precursors are conflicting (81), which may complicate the design of MC2R antagonists that also block ACTH precursors.

### A Specific MC2R Antagonist: An Elusive Target

In the last four decades, extensive studies that have been performed to determine the molecular basis of the interaction of ACTH with cognate MCRs have shown inconsistent results, mainly due to technical limitations. First, the expression at the cell membrane of human MC2R in cell lines was not possible until the discovery of the melanocortin receptor-associated protein (MRAP) (46), which is required to address MC2R at the cell membrane. Second, studies on putative ACTH antagonists failed to perform a systematic analysis of ACTH antagonists’ activation and binding against all cognate MCRs to assess specificity (83, 84).

Melanocortin receptors belong to the GPCRs family that has natural agonists and antagonists. The melanocortin antagonists, agouti-related protein (AGRP) and agouti signaling protein (ASIP), are the only two endogenous antagonists of MCRs identified to date (85–87). The primary sequences of the endogenous agonists (MSHs) and antagonists (AGRP and ASIP) are different, and they interact differently with the MCRs to produce the active and inactive conformations of the ligand–receptor complex.

The design of a specific peptide remains a challenge that has not been resolved because of the difficulty of designing a peptide specific for one MCR (e.g., MC2R) (37). Indeed, all melanocortin ligands receptors shared the same H_6_F_7_R_8_W_9_ motif, which is important for MCR binding and stimulation (88) and which bind to all MCR receptors. Development of selective ligands for the melanocortin system is challenging due to the conserved amino acid sequences of the MCRs and of their structural similarity in the seven-transmembrane GPCR fold (89). The limited structural variations of the endogenous melanotropin ligands further reduce options in the design of MCR ligands for achieving selectivity. If they are not selective, these peptides could be the source of severe undesirable effects due to the numerous specific roles of the five MCRs. Of note, some ACTH fragments show variable specificity across species. For example, ACTH (7–38) fragment antagonizes the human MC2 receptor but stimulates aldosterone secretion from the rat adrenal cortex through a mechanism involving the angiotensin receptor (90). Variable specificity between species is therefore another challenge for the preclinical phase of the development of new ACTH receptor agonists. A table listing MC2R agonists and antagonists is available in Table S1 in Supplemental Material.

Designing molecules that possess both functional selectivity and human melanocortin receptor (hMCR) subtype selectivity from the melanotropin core sequence H_6_F_7_R_8_W_9_ has been difficult. Many peptides are conformationally flexible in aqueous solution, but upon interacting with their biologically relevant molecule, they assume a preferred conformation. The reduction of conformational freedom may eventually lead to the receptor bound conformation, which results in the selective interaction of a ligand with a receptor. A major step is to determine which peptide conformation is required for binding to the receptor and resulting in an agonist or antagonist effect. A critical approach is to understand if changes in ACTH three-dimensional (3D) conformation are associated with antagonist properties (Figure 2). X-ray crystal structures provide 3D conformation but may be misleading in terms of function. Incorporation of these X-ray coordinates into a computer-aided examination of function into 3D space is being pursued (91). This allows one to further explore the region/site or the surrounding 3D space occupied by the key amino acids of the protein (where the potent ligand has an affinity) so as to better understand biological actions. Nevertheless, the numerous physiological functions of the five known subtypes of hMCRs continue to be a stimulus for the production of selective melanocortin agonists and antagonists (92).

## Conclusion

The ACTH is the pituitary hormone that allows growth and development of the adrenal cortex and glucocorticoid synthesis. Even if the structure of ACTH has been known for decades, the exact mechanism of activation of MC2R, the specific ACTH receptor on adrenal cells, is still unknown. Understanding this activation and the development of specific MC2R antagonists would allow the treatment of some difficult-to-treat diseases such as CAH or PBMAH.

## Author Contributions

CG wrote the review and revised the final version; J-MV performed bioinformatic analysis and revised the final version; and JD wrote the review and revised the final version.

## Conflict of Interest Statement

The authors declare that the research was conducted in the absence of any commercial or financial relationships that could be construed as a potential conflict of interest.

## Abbreviations

ACTH, adrenocorticotropin; PBMAH, primary bilateral macronodular adrenal hyperplasia; AGRP, agouti-related protein; ASIP, agouti signaling protein; CAH, congenital adrenal hyperplasia; FGD, family glucocorticoid deficiency; MSH, melanocyte-stimulating hormone; MCR, melanocortin receptor; MRAP, melanocortin receptor-associated protein; POMC, proopiomelanocortin.
