# Supplementary material for: From Bioinactive ACTH to ACTH Antagonist: The Clinical Perspective
Source: Front Endocrinol (Lausanne). 2017 Feb 8;8:17. doi: 10.3389/fendo.2017.00017 (PMC5296294; doi:10.3389/fendo.2017.00017)
Supplement: Supplementary file 1 [file table_1.docx]

| Molecule | Species | MC2R Agonist | MC2R Antagonist |
| --- | --- | --- | --- |
| ACTH (7-38) (CIP, cortricotropin inhibiting peptide)^1^ | Rat | ø | Competitive antagoniste of ACTH(1-39) |
| ACTH (11-24) ^2,3^ | Frog | Dose related stimulation of cortico and aldosterone ( from 3.16x10^-8^M lower dose to 3.16x10^-10^M half maximum effect) | Competitive antagoniste of ACTH(1-39) and of ACTH(1-10)^3,4^ |
| ACTH(1-24 )_2_ Lys - dimer ^2^ | Frog | Weak induction of corticosterone and aldosterone (70 times less potent that ACTH 1-24 or ACTH1-39) | ø |
| ACTH(1-24)^2^ | Frog | Agonist | ø |
| ACTH(1-39)^2^  (10^-9^M) | Frog | Dose related stimulation of cortico and aldosterone ( from 3.16x10^-11^M lower dose to 3.16x10^-10^M half maximum effect) | ø |
| ACTH(11-24)_2_ Lys- dimer ^2^ ^3^ | Frog | ø | Reduced ACTH (1-39) evoked stimulation of corticosterone and aldosterone release(63 and 62 %respectively) |
| ACTH Glu(11-24)_2_ - dimer  ^3,4^ | Frog | ø | Antagonist |
| ACTH(5-24)  ^5^ | Rat | Full agonist | ø |
| ACTH(6-24)  ^6^ | Rat | ø | Competitive inhibitor of ACTH (1-39) |
| ACTH(6-39)  ^7^ | Rat | ø | Competitive inhibitor of ACTH (1-39) |
| ACTH(1-24)  ^8^ | Rat | Full agonist and as potent as ACTH(1-39) | ø |
| ACTH(5-24) ^9,10^ | Bovine^9^  Rat^10^ | Partial agonist  Full lipolytic agonist | ø |
| ACTH (6-24)^9^ | Bovine | Partial agonist | ø |
| ACTH(7-24)^9,10^ | Bovine^9^  Rat^10^ | Partial agonist  Do not stimuated lipolysis | ø |
| AGRP and agouti protein^11^ | Human | ø | MC4R natural antagonist |
| ACTH(8-39)^12^ | Human | ø | Partial antagonist (CIP) |
| ACTH(15-18)^13^ | Human | ø | Competitive antagonist of ACTH receptor |
| ACTH W9Y^14^ | Bovine | ø | Antagonist |
| ACTH(15-24)^15,16^  Dores RM. *ACTH Antagonist Peptides*. US Patent application US 2012/0309696 A1 (2012). | RAT  Mammalian  OS3 adrenal cell line transfected with a hmc2R cDNA | ø | Antagonist |
| GPS1573, a variant of ACTH (7-18) with an N terminal nor leucine-proline sequence and D-Phe and DD-Trp (in place of L-Phe, L-Trp) in the HFRW sequence^17,18^ | HEK293 cells (ATCC, CRL-1573) were stably transfected with human MC2R and MRAP cDNAs^17^  Rat^18^ | ø | Antagonist |
| GPS1574, a cyclized variant of GPS1573^17,18^ | HEK293 cells (ATCC, CRL-1573) were stably transfected with human MC2R and MRAP cDNAs^17^  Rat^18^ | ø | Has alos antagonist effect on MC3R,MC4R and MC5R |
| IRC-274^19^ | HEK293 cells (ATCC, CRL-1573) were stably transfected with human MC2R and MRAP cDNAs | ø | Selective MC2R antagonist |
| -melanotan II (MTII)  -tetrapeptides  -MK-0489  -MK-0493  -urea-based piperazine  -Ro27-3225  -Cyclophanes  -ACTH(1-13) (α MSH)  -compound 1  -pyrrolidine diastereoisomer  -BIMs (BIM-22493 and BIM-22511)  -β-MSH analogues  ^20^ | -Rat  -Rat  -Mice  -Human  -Rat  -Rat  -Rat  -Rat  -Mice  -HEK293 cells expressing  MC4R  - CHO-K1 cells transfected with MCR  -Rat | MC4R agonist | ø |
| Corticostation or defensin α 4^21^ | Rat (isoleted from rabbit lung but tested on rat) | ø | Non specific MC2R antagonsit |
| ACTH (4-10)^22^ | HEK cells that expressed either the rat melanocortin MC3 receptor, the human melanocortin MC4 receptor or the ovine melanocortin MC5 receptor | ø | MC3R and MC4R antagonist |
| SEMAX^23^ |  | ø | MC4R antgonist |
| 153N6^24^ | Frog | ø | MC1R antagonist but bind to MC3R,MC4R and MC5R with low affinity |
| [D-Trp7,D-Phe10]a-MSH (6 –11)amide^25^ | Mouse melanoma cells (which express the native receptor MC1) and MSH unresponsive human kidney 293 cells transfected with the expression vector pcDNA 1Neo | ø | MC3R antagonist |
| ACTH(1-17)^26,27^ | OS3 adrenal cell line ^26^  Hela cell line transfected with the cloned mouse adrenocorticotropin receptor expressed | Agonist (?)  But limited activity due to the lack of KKRRP sequence ^27^. | ø |
| ACTH (1-16)^26^ | OS3 adrenal cell line | Agonist (?)  But limited activity due to the lack of KKRRP sequence. | ø |
| NDP-MSH ([Nle4, DPhe7]-a-MSH)  ^28^ | Human | non-selective  agonist for the human MC1, MC3, MC4 and MC5 receptors | ø |
| Melanotan II (MTII, the  lactam Ac-Nle4 cycle [Asp-His6-DPhe7-Arg8-Trp9-Lys10]-  amide)  ^28^ | The coding regions of the genes for the hMCRs wild-type, and  mutants were subcloned into the pCDNA3.1. HEK-293 cell line  was purchased from ATCC | non-selective  agonist for the human MC1, MC3, MC4 and MC5 receptors | ø |
| SHU9119 : Substitution of a bulky hydrophobic amino acid at the  Phe position  ^28-30^ | -The coding regions of the genes for the hMCRs wild-type, and  mutants were subcloned into the pCDNA3.1. HEK-293 cell line  was purchased from ATCC  - human and mouse  -prostate cancer cell | ø | converts the MTII peptide from an  agonist into an antagonist at the MC3 and MC4 receptors |
| SHU8914^29,31^ | Human and mouse | ø | full agonists of the MC1-R and  MC5-R, weak partial agonists of hMC3-R and subsequently characterized  as potent antagonists of the hMC3-R  as well as the hMC4R |
| HS024^32^ | goldfish | ø | MC4R antagonist |
| Substitution of  tryptophan by phenylalanine or by Nα-methyltryptophan  as in [Gln^5^, Phe^9^]ACTH 1-20 amide or [Nα-Metrp^9^ ]ACTH l-24^14^ | Bovine cortical adrenal membrane |  | MC2R antagonist:  provides ACTH analogs that exhibit high  affinity for the ACTH receptor(s) but fail to activate the adenylate cyclase system |
| AC-Nle-Asp-Trp-D-Phe-Nle-Trp-Lys-NH2^33^ | Mice  human | ø | MC1R antagonist with agonist effect on MC3R, MC4R and MC5R |
| c[Gly-Cpg-D-Nal(2’)-Arg-Trp-Glu]-Val-Val-Gly-NH_2_^34^ | melanocortin-1 (MC1) receptor from Xenopus frog skin    recombinant human MC1, MC3, and MC4 receptors expressed in human embryonic kidney (HEK) cells | ø | MC1R antagonist but highly selective versus human MC4R and modestly selective versus MC3R |
| N-methyltryptophan instead of Trp at position 9 of ACTH 1-24^14^ | Bovine | ø | MC2R antagonist |
| Synthetic peptide GKVLKKRR (fragments 81-88 of pro IL 1α protein)^35^ | Rat | ø | MC2R antagonist |
| [D-Trp^8^]γ-MSH^36^ | WT C57BL/6J mice  MC3RKO mice and WT | ø | hMC3R selective agonist |
| ACTH(1-18)  Three  modifications (DSer was introduced instead of Ser on the  position 1, the sequence 15-18 was changed to Lys-Lys-Lys-  Lys and the C-terminus was amidated)^8^ | Rat | Full agonist, 5 fold more potent in vivo than ACTH(1-39) | ø |
| ACTH (1-23)^8^ | Rat | Full agonist | ø |
| ACTH (1-10)-(15-19)^37^ | Rat |  | ø |
| ACTH (7-39)^27^ | Hela cell line transfected with the cloned mouse adrenocorticotropin receptor expressed |  | Effective MC2R antagonist when used at 100 fold molar excess |
| ACTH(4-23)(NH_2_)^38^ | Rat | High activity but less potent than ACTH (1-24) |  |
| ACTH (5-23)^38^ | Rat | Less potent than ACTH (1-24) but induces corticosterone stimulation | ø |
| ACTH(6-24)^38^ | Rat | Low activity | ø |
| ACTH(1-10)^38^ | Rat | High activity in vivo and in vitro (stimulates corticosterone production) | ø |
| GPS1573^18^ | Rat | In vivo : lead to a small corticosterone response to ACTH | In vitro MC2R antagonist^17^ |
| GPS1574^18^ | Rat | Inhibition of corticosterone response to ACTH | In vitro MC2R antagonist^17^ |
| Substitution of Phe^7^ with D-Nal(2’) in ACTH(1–24)  ^39^ | OS3 and HEK cell lines, lacking endogenous MC2R, were used for hMC4R, hMC2R, and chimeric receptor transfection | did not switch the ligand from agonist to antagonist at  MC2R, which was observed inMC3Rand MC4R |  |

1 Malendowicz, L. K., Rebuffat, P., Nussdorfer, G. G. & Nowak, K. W. Corticotropin-inhibiting peptide enhances aldosterone secretion by dispersed rat zona glomerulosa cells. *J Steroid Biochem Mol Biol* **67**, 149-152 (1998).

2 Feuilloley, M., Stolz, M. B., Delarue, C., Fauchere, J. L. & Vaudry, H. Structure-activity relationships of monomeric and dimeric synthetic ACTH fragments in perifused frog adrenal slices. *J Steroid Biochem* **35**, 583-592 (1990).

3 Fauchere, J. L., Rossier, M., Capponi, A. & Vallotton, M. B. Potentiation of the antagonistic effect of ACTH11-24 on steroidogenesis by synthesis of covalent dimeric conjugates. *FEBS Lett* **183**, 283-286 (1985).

4 Seelig, S., Sayers, G., Schwyzer, R. & Schiller, P. Isolated adrenal cells: ACTH(11-24), a competitive antagonist of ACTH(1-39) and ACTH(1-10). *FEBS Lett* **19**, 232-234 (1971).

5 Seelig, S., Lindley, B. D. & Sayers, G. A new approach to the structure-activity relationship for ACTH analogs using isolated adrenal cortex cells. *Methods Enzymol* **39**, 347-359 (1975).

6 Bristow, A. F., Gleed, C., Fauchere, J. L., Schwyzer, R. & Schulster, D. Effects of ACTH (corticotropin) analogues on steroidogenesis and cyclic AMP in rat adrenocortical cells. Evidence for two different steroidogenically responsive receptors. *Biochem J* **186**, 599-603 (1980).

7 Ways, D. K., Mahaffee, D. D. & Ontjes, D. A. An adrenocorticotropin analog [ACTH(6-39)] which acts as a potent in vitro adrenocorticotropin antagonist at low calcium concentration and as a weak agonist at high calcium concentration. *Endocrinology* **104**, 1028-1035, doi:10.1210/endo-104-4-1028 (1979).

8 Schwyzer, R. ACTH: a short introductory review. *Ann N Y Acad Sci* **297**, 3-26 (1977).

9 Bonnafous, J. C., Fauchere, J. L., Schlegel, W. & Schwyzer, R. Hormone-receptor interactions. Stimulation and inhibition of bovine adrenal cortex cell membrane adenylate cyclase by synthetic corticotropin fragments and the effect of 5'-guanylylimidodiphosphate. *FEBS Lett* **78**, 247-250 (1977).

10 Lang, U., Fauchere, J. L., Pelican, G. M., Karlaganis, G. & Schwyzer, R. Hormone-receptor interactions. Adrenocorticotrophin-(7-24)-octadecapeptide stimulates adipocyte membrane adenylate cyclase without causing lipolysis in fat cells. *FEBS Lett* **66**, 246-249 (1976).

11 Clark, A. J. *et al.* ACTH Antagonists. *Front Endocrinol (Lausanne)* **7**, 101, doi:10.3389/fendo.2016.00101 (2016).

12 Li, C. H., Chung, D., Yamashiro, D. & Lee, C. Y. Isolation, characterization, and synthesis of a corticotropin-inhibiting peptide from human pituitary glands. *Proc Natl Acad Sci U S A* **75**, 4306-4309 (1978).

13 Kovalitskaia Iu, A. *et al.* [Synthetic peptide KKRR corresponding to the human ACTH fragment 15-18 is an antagonist of the ACTH receptor]. *Bioorg Khim* **34**, 29-35 (2008).

14 Hofmann, K., Montibeller, J. A. & Finn, F. M. ACTH antagonists. *Proc Natl Acad Sci U S A* **71**, 80-83 (1974).

15 Dores, R. M. ACTH Antagonist Peptides. doi:0309696 A1 (2012).

16 Liang, L., Angleson, J. K. & Dores, R. M. Using the human melanocortin-2 receptor as a model for analyzing hormone/receptor interactions between a mammalian MC2 receptor and ACTH(1-24). *Gen Comp Endocrinol* **181**, 203-210, doi:10.1016/j.ygcen.2012.11.011 (2013).

17 Bouw, E. *et al.* Development of potent selective competitive-antagonists of the melanocortin type 2 receptor. *Mol Cell Endocrinol* **394**, 99-104, doi:10.1016/j.mce.2014.07.003 (2014).

18 Nensey, N. K., Bodager, J., Gehrand, A. L. & Raff, H. Effect of Novel Melanocortin Type 2 Receptor Antagonists on the Corticosterone Response to ACTH in the Neonatal Rat Adrenal Gland In Vivo and In Vitro. *Front Endocrinol (Lausanne)* **7**, 23, doi:10.3389/fendo.2016.00023 (2016).

19 Halem HA, U. M., Jewett I, Bastille A, Beech J, et al. In vivo

suppression of corticosterone in rodent models of Cushing’s disease with a selective, peptide MC2 receptor antagonist. (2016).

20 Fani, L., Bak, S., Delhanty, P., van Rossum, E. F. & van den Akker, E. L. The melanocortin-4 receptor as target for obesity treatment: a systematic review of emerging pharmacological therapeutic options. *Int J Obes (Lond)* **38**, 163-169, doi:10.1038/ijo.2013.80 (2014).

21 Zhu, Q. Z. *et al.* Isolation and structure of corticostatin peptides from rabbit fetal and adult lung. *Proc Natl Acad Sci U S A* **85**, 592-596 (1988).

22 Adan, R. A. *et al.* Identification of antagonists for melanocortin MC3, MC4 and MC5 receptors. *Eur J Pharmacol* **269**, 331-337 (1994).

23 Ashmarin, J. P. N., V. N.; Levitskaya, N. G.; Koshelev, & V. B.; Kamensky, A. A. Design and investigation of an ACTH (4 –10)

analogue lacking D-amino acids and hydrophobic residues. *neuroscience* **16**, 105–112 (1995).

24 Jayawickreme, C. K., Quillan, J. M., Graminski, G. F. & Lerner, M. R. Discovery and structure-function analysis of alpha-melanocyte-stimulating hormone antagonists. *J Biol Chem* **269**, 29846-29854 (1994).

25 Tatro, J. B. & Entwistle, M. L. Identification of a specific mammalian melanocortin receptor antagonist. *Ann N Y Acad Sci* **739**, 315-319 (1994).

26 Chen, M., Aprahamian, C. J., Kesterson, R. A., Harmon, C. M. & Yang, Y. Molecular identification of the human melanocortin-2 receptor responsible for ligand binding and signaling. *Biochemistry* **46**, 11389-11397, doi:10.1021/bi700125e (2007).

27 Kapas, S., Cammas, F. M., Hinson, J. P. & Clark, A. J. Agonist and receptor binding properties of adrenocorticotropin peptides using the cloned mouse adrenocorticotropin receptor expressed in a stably transfected HeLa cell line. *Endocrinology* **137**, 3291-3294 (1996).

28 Chen, M., Georgeson, K. E., Harmon, C. M., Haskell-Luevano, C. & Yang, Y. Functional characterization of the modified melanocortin peptides responsible for ligand selectivity at the human melanocortin receptors. *Peptides* **27**, 2836-2845, doi:10.1016/j.peptides.2006.04.012 (2006).

29 Hruby, V. J. *et al.* Cyclic lactam alpha-melanotropin analogues of Ac-Nle4-cyclo[Asp5, D-Phe7,Lys10] alpha-melanocyte-stimulating hormone-(4-10)-NH2 with bulky aromatic amino acids at position 7 show high antagonist potency and selectivity at specific melanocortin receptors. *J Med Chem* **38**, 3454-3461 (1995).

30 Hafiz, S. *et al.* Expression of melanocortin receptors in human prostate cancer cell lines: MC2R activation by ACTH increases prostate cancer cell proliferation. *International journal of oncology* **41**, 1373-1380, doi:10.3892/ijo.2012.1574 (2012).

31 Irani, B. G. *et al.* Progress in the development of melanocortin receptor selective ligands. *Curr Pharm Des* **10**, 3443-3479 (2004).

32 Cerda-Reverter, J. M., Ringholm, A., Schioth, H. B. & Peter, R. E. Molecular cloning, pharmacological characterization, and brain mapping of the melanocortin 4 receptor in the goldfish: involvement in the control of food intake. *Endocrinology* **144**, 2336-2349, doi:10.1210/en.2002-0213 (2003).

33 Mogil, J. S. *et al.* The melanocortin-1 receptor gene mediates female-specific mechanisms of analgesia in mice and humans. *Proc Natl Acad Sci U S A* **100**, 4867-4872, doi:10.1073/pnas.0730053100 (2003).

34 Han, G., Quillan, J. M., Carlson, K., Sadee, W. & Hruby, V. J. Design of novel chimeric melanotropin-deltorphin analogues. Discovery of the first potent human melanocortin 1 receptor antagonist. *J Med Chem* **46**, 810-819, doi:10.1021/jm020355o (2003).

35 Vanina, V. I. *et al.* [Stress-protective effect of the synthetic ACTH-like peptide leucocorticotropin]. *Bioorg Khim* **32**, 477-484 (2006).

36 Marks, D. L., Hruby, V., Brookhart, G. & Cone, R. D. The regulation of food intake by selective stimulation of the type 3 melanocortin receptor (MC3R). *Peptides* **27**, 259-264, doi:10.1016/j.peptides.2005.01.025 (2006).

37 L CHOH Hao, L. R., J; Chung, D. Syntheis of a Biologically Active

Pentadecapeptide Corresponding to an Altered Sequence of the

Adrenocorticotropin (ACTH) Structure J Am Chem Soc. *Journal of the American Chemical Society* **86**, 2711-2715, doi:10.1021/ja01067a039 (1964).

38 Sayers, G. *et al.* Isolated adrenal cortex cells: ACTH4-23 (NH2), ACTH5-24, ACTH6-24 and ACTH7-23 (NH2); cyclic AMP and corticosterone production. *Proc Soc Exp Biol Med* **145**, 176-181 (1974).

39 Yang, Y. *et al.* Third transmembrane domain of the adrenocorticotropic receptor is critical for ligand selectivity and potency. *J Biol Chem* **290**, 7685-7692, doi:10.1074/jbc.M114.596122 (2015).
